# Molecular Epidemiology of SARS-CoV-2 within Accra Metropolis Postlockdown

**DOI:** 10.1155/2024/2993144

**Published:** 2024-03-29

**Authors:** Frank T. Aboagye, Lawrence Annison, Henry Kwadwo Hackman, Maame E. Acquah, Yvonne Ashong, Isaac Owusu-Frimpong, Bill C. Egyam, Sharon Annison, George Osei-Adjei, Samuel Antwi-Baffour

**Affiliations:** ^1^Department of Medical Laboratory Technology, Faculty of Applied Sciences, Accra Technical University, Accra, Ghana; ^2^Biomedical and Public Health Research Unit, Council for Scientific and Industrial Research–Water Research Institute, Accra, Ghana; ^3^West African Centre for Cell Biology of Infectious Pathogens, College of Basic and Applied Sciences, University of Ghana, Legon, Accra, Ghana; ^4^Department of Parasitology, Noguchi Memorial Institute of Medical Research, College of Medical Sciences, University of Ghana, Legon, Accra, Ghana; ^5^Department of Molecular Biology, MDS Lancet Laboratories Ghana Limited, East Legon, Accra, Ghana; ^6^Department of Epidemiology and Disease Control, School of Public Health, University of Ghana, Legon, Accra, Ghana; ^7^Department of Medical Laboratory Sciences, School of Biomedical and Allied Health Sciences, College of Health Sciences, University of Ghana, Korle-Bu, Accra, Ghana

## Abstract

**Introduction:**

Currently, sequencing has been the only tool for the identification of circulating severe acute respiratory syndrome coronavirus-2 (SARS-CoV-2) variants. However, it is known to be an expensive and laborious approach involving high technical expertise. Considering the reduced adherence to preventive measures postlockdown in Accra, this study presents an alternative method that leverages polymerase chain reaction (PCR) to identify circulating SARS-CoV-2 variants in the Accra Metropolis postlockdown.

**Methods:**

This prospective cross-sectional study was conducted between July and December 2022. Nasopharyngeal samples were collected from 268 consenting participants. Samples were subjected to nucleic acid extraction and followed by real-time polymerase chain reaction for the detection and quantification of SARS-CoV-2 RNA. SARS-CoV-2 positive samples were subsequently subjected to variant identification using rapid PCR. *Findings*. The prevalence of SARS-CoV-2 within the Accra Metropolis was 30.2%. The majority of the SARS-CoV-2 infection was diagnosed in females, participants aged 41–50 years, and symptomatic participants. Participants aged ≤10 years and females recorded the highest viral load while participants aged 41–50 years recorded the highest number of infections. The SARS-CoV-2 variants detected were Alpha (64.2%), Delta (22.2%), and Omicron (13.6%). Predictors of SARS-CoV-2 infection identified were chills, cough, headache, body weakness, sore throat, and dyspnoea in order of decreasing association with SARS-CoV-2 infection. There was a strong association between symptom status, gender, age, and SARS-CoV-2 infection.

**Conclusion:**

There was a high prevalence of SARS-CoV-2 within the Accra Metropolis postlockdown within the sampling period. The Alpha variant of SARS-CoV-2 is the predominant circulating variant, and persons presenting with symptoms are most likely to be diagnosed with COVID-19. Children aged ≤10 years serve as a reservoir for infection transmission.

## 1. Introduction

The first case of coronavirus disease-2019 (COVID-19) caused by the severe acute respiratory syndrome coronavirus-2 (SARS-CoV-2) was reported in Wuhan, China, in December 2019 [1]. Ghana recorded its first two cases of COVID-19 on March 12, 2020, which were imported from Norway and Turkey [2]. As of October 22, 2021, there have been over 200 million confirmed cases of COVID-19 and more than 4 million deaths across 187 countries. In Ghana, there have been 171,023 confirmed cases and 1,461 deaths as of December 13, 2022 [3]. While the spread of SARS-CoV-2 in Africa has not been alarming compared to other continents, several reports have shown that adults aged 60 years and above have a high risk of mortality regardless of their geographic location [4, 5].

COVID-19 is transmitted through contact with droplets from an infected person when they cough, sneeze, or talk. Although over 50% of cases remain asymptomatic [6, 7], COVID-19 is typically characterized by symptoms such as fever, headache, sore throat, dyspnoea, muscle aches, general weakness [8, 9], and in some cases, gastrointestinal symptoms including diarrhoea [10, 11].

In an effort to combat the spread of COVID-19 in major cities throughout Ghana, the government implemented several measures including lockdowns, border closure, and a ban on social gatherings such as weddings, funerals, and church services. These interventions were put into effect on March 30, 2022. While data on the molecular epidemiology of the disease in Ghana have been scarce following the lockdown, several studies have been conducted in order to explore the disease's epidemiological profile on a larger scale through surveillance and contact tracing [2, 12–14]. Evidence from Kenu et al. [14] suggests that adherence to the preventive measures during the lockdown resulted in a decline in COVID-19 cases due to the adherence of the populace to the preventive measures.

Postlockdown studies on the molecular epidemiology of SARS-CoV-2 within the Accra Metropolis have been limited. Currently, sequencing is the only tool available for identifying circulating SARS-CoV-2 variants, and it is known to be expensive and requires significant technical expertise. Given the reduced adherence to preventive measures postlockdown in Accra and other sub-Saharan African countries [15], this study presents an alternative method for identifying circulating SARS-CoV-2 variants in the Accra Metropolis postlockdown. This alternative approach leverages PCR, which is both less expensive and less laborious than sequencing.

## 2. Materials and Methods

### 2.1. Ethics Statement

The study protocol (Protocol no. ATU/MLT/ET/01192304B/2021-2022) was approved by the Ethical Review Committee of the Medical Laboratory Technology Department, Accra Technical University. Informed and written consent was obtained from participants aged 18 years and above. Assent was obtained from parents or guardians on behalf of participants below the age of 18 years. Permission was granted by MDS-Lancet Laboratories Ghana Limited before the study was carried out. Study participants were assured of the strict confidentiality and safety of any information they provided for the study.

### 2.2. Study Area

This study was conducted in the Accra Metropolis at the MDS-Lancet Laboratories Ghana Limited located at East Legon, Accra, Ghana, from July to December 2022. The COVID-19 laboratory of the MDS-Lancet Laboratories Ghana Limited was established in 2020 during the early stages of the pandemic and receives samples from satellite laboratories across the country, including many from within the Accra Metropolis. MDS-Lancet Laboratory is an ISO-certified (ISO/IEC 15189: 2012) medical diagnostic laboratory with over 20 branches throughout Ghana.

### 2.3. Study Design and Sample Collection

The study was a cross-sectional study. A convenient sampling method was employed to enroll participants in the study. Study participants were chosen from among individuals who voluntarily walked into the facility to be tested. A nasopharyngeal sample was taken from each study participant, and the swabs were placed into a viral transport medium (Shanghai Escusgen Biotechnology Co., Ltd, China) in a cold chain (4°C–8°C) and sent to the laboratory for analysis. Samples were collected from participants presenting with symptoms as well as participants requesting COVID-19 tests for travelling and routine testing.

## 3. Laboratory Analysis

### 3.1. Nucleic Acid Isolation

RNA was extracted from each nasopharyngeal specimen using the Zymo™ Quick Viral RNA Extraction kit (Zymo™ Research Cooperation, USA) following the manufacturer's  instructions with some modifications as described by Aboagye and Acquah [16].

### 3.2. Real-Time Polymerase Chain Reaction (RT-PCR)

The amplification of the SARS-CoV-2 RNA was performed using the Allplex 2019n-CoV amplification Kit (Seegene Inc., Korea) following the manufacturer's protocol. The amplification was performed on CFX 96 1000 series Thermocycler (Bio-Rad, USA) with thermal conditions specific to the Allplex™ 2019-n-CoV amplification kit (Seegene  Inc., Korea) and preparation of the reaction mix as described elsewhere [16]. Quantification of viral loads was performed using serial dilutions of the positive control provided with the amplification kit to develop a standard curve for extrapolation of the viral loads of each SARS-CoV-2 positive sample.

All samples with a cycle threshold (Ct) of 40 and above were considered negative for SARS-CoV-2 infection. The assay was validated with the addition of negative control and positive control. The Allplex™ 2019-n-CoV Assay Kit detects three viral genes (N, RdRp, and E). Sample positivity was determined with the following criteria: N, RdRp, and E genes amplified with or without the presence of the internal control (IC) and positive if both the N gene and RdRp were amplified.  If only the E gene was amplified,  it was considered as a presumptive positive, thus requiring assay repetition [17].

### 3.3. SARS-CoV-2 Variant Identification

Variant identification was performed using the Allplex SARS-CoV-2 Variant II Assay in a 20 *μ*l reaction according to the manufacturer's instruction. In brief, 15 *μ*l of the Allplex SARS-CoV-2 Variant II PCR mix and 5 *μ*L of SARS-CoV-2 RNA were loaded into each well. The thermocycling was performed as described on a CFX 96 1000 series Thermocycler (Bio-Rad Laboratories, USA) following cycling conditions described by Umunnakwe et al. [18]. The Allplex SARS-CoV-2 Variant II Assay detects four mutations in the S gene, the W152C mutation, K417T mutation, K417N mutation, and L452R mutation, using the HEX, Cal Red 610, Quasar 705, and FAM fluorescent dyes, respectively. The Allplex SARS-CoV-2 Variant II Assay uses an endogenous internal control, which is detected using the Quasar 670 fluorescent signal channel. The results are automatically analysed using the SARS-CoV-2 Viewer V1 Trial Variant II Software (Seegene Inc., Republic of Korea) and interpreted as described in Lotti et al. [19]. The Allplex SARS-CoV-2 Variant II Assay has been validated in previous studies reporting an agreement of 100% (CI_95_: 96.7–100.0) with Whole Genome Next Generation Sequencing [18, 20, 21].

### 3.4. Quality Control

The integrity of samples collected was ensured following established sample collection and transportation guidelines for nasopharyngeal specimens for SARS-CoV-2 testing [22, 23]. Samples collected were transported to the laboratory at temperatures between 4 and 8°C in triple packaging. RNA was extracted from samples within two hours of collection and amplification was performed immediately after extraction. The study also included an MS2 Phage full-process internal control that is not subject to variations in the human genomic material to validate the extraction and PCR process. In each PCR, the study included a positive and negative control, which was provided by the manufacturer of the amplification kit. Nuclease-free water was extracted and amplified along with each batch of samples as a negative process control. This was performed to maintain the integrity of the experimental process, detect contamination, and ensure the reliability and validity of the results obtained.

### 3.5. Statistical Analysis

Data were entered into Microsoft Office Excel 2019 and imported into Statistical Package for the Social Sciences (SPSS) version 27 (IBM, USA) and GraphPad Prism 9.0 (GraphPad, San Diego, CA, USA) for analysis. For continuous and categorical variables, descriptive statistics were computed. For data without normal distribution, median and interquartile range (IQR) were computed, while mean with a 95% confidence interval was computed for normally distributed data. For categorical variables, percentages were also calculated. Statistical comparison between subgroups of categories was evaluated by *t*-test, analysis of variance (ANOVA) and chi-square test where appropriate. Multivariate analysis was performed to explore the association between infection status and demographic characteristics as risk factors and the reported symptoms as predictors.

## 4. Results

### 4.1. Characteristics of Study Participants and Clinical Presentations

The present study involved a total of 268 participants of which the majority were males 51.5% (*n* = 138, 51.5%). When stratified according to age, study participants aged 31–40 years formed the majority of the study population (*n* = 51, 19.0%), while participants aged 70 years and above were the least in numbers (*n* = 17, 6.3%). About 213 (79.5%) of the study participants were Ghanaians and 20.5% (*n* = 55) of the study participants were non-Ghanaians as shown in Table 1.

**Figure 1:**
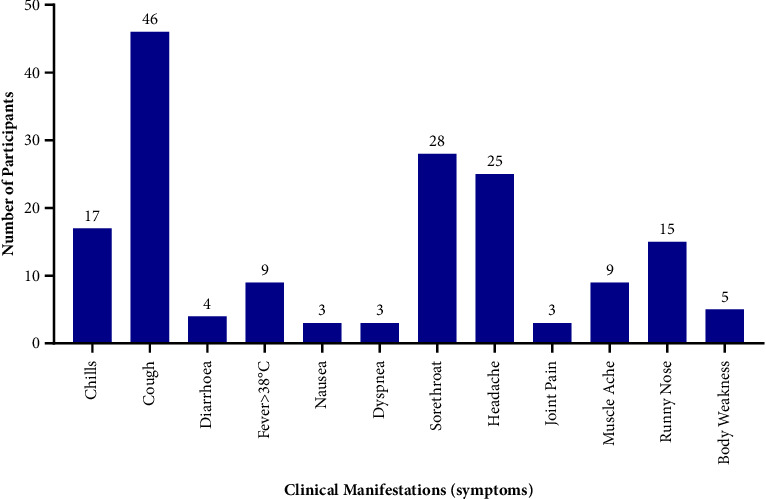
SARS-CoV-2-associated symptoms presented by study participants.

Figure 1 gives a detailed description of the symptoms presented by the study participants. This included fever, shortness of breath, headache, and sore throat amongst others. Participants who reported at least one of these symptoms were 55 (20.5%), and 213 (79.5%) of the participants were asymptomatic. As shown in Figure 1, nausea, dyspnoea, and joint pains were the least reported symptoms.

### 4.2. Prevalence of SARS-CoV-2 Infection

The prevalence of SARS-CoV-2 infection reported in this study was 30.20% (C I_95_: 29.57–30.89).  Table 2 gives a detailed description of the distribution of SARS-CoV-2 in relation to the demographic characteristics of the study participants. The study reported a high prevalence of SARS-CoV-2 in females (56.8%) as well as in participants aged 31–40 years (24.7%). Furthermore, the majority of the SARS-CoV-2 cases reported in this study were detected in symptomatic participants (53.1%) as well as Ghanaian participants (76.5%) as shown in Table 2. There was no statistically significant association between SARS-CoV-2 infection status and age, gender, and nationality (*p* > 0.05). However, a significant association was observed between SARS-CoV-2 infection status and being symptomatic (*p* < 0.001).

### 4.3. Severity of SARS-CoV-2 Infection

**Figure 2:**
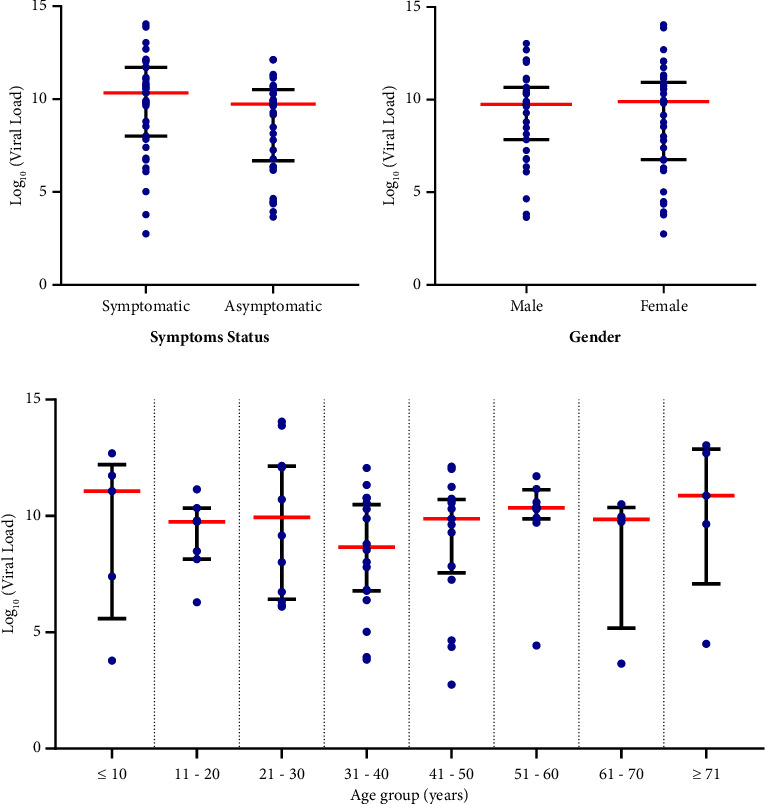
Distribution of SARS-CoV-2 viral load among study participants: (a) SARS-CoV-2 viral load stratified by symptomatic status, (b) SARS-CoV-2 viral load stratified by gender, and (c) SARS-CoV-2 viral load stratified by age groups.

The study quantified the copies of SARS-CoV-2 RNA in each of the 81 positive cases. The overall median viral load was 2.1 × 10^9^ copies/*μ*l (IQR: 5.7 × 10^6^–2.3 × 10^10^).  Figure 2 gives a detailed description of the distribution of SARS-CoV-2 viral load amongst study participants. Female participants had a higher viral load (2.70 × 10^9^ copies/*μ*l, IQR: 1.3 × 10^6^–2.3 × 10^10^), while symptomatic participants reported even higher viral loads (5.5 × 10^9^ copies/*μ*l, IQR: 1.9 × 10^7^–2.1 × 10^11^). Nonetheless, there was no statistically significant difference between the median viral load of symptomatic and asymptomatic participants (*p*=0.074) or between male and female participants (*p*=0.996). Participants aged ≤10 years had the highest median viral load (4.5 × 10^10^ copies/*μ*l, IQR: 3.1 × 10^6^–5.7 × 10^11^) (Figure 2(c)), but no significant difference was found in median viral loads across age groups (*p* > 0.05). As shown in Figure 2, the correlation analysis revealed that there was an insignificant inverse relationship between viral load and age (*r* = −0.117, *p*=0.279).

**Figure 3:**
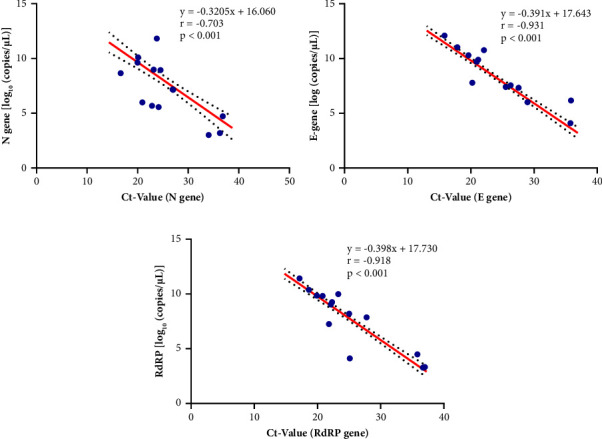
Association between viral load and Ct-values: (a) N gene viral load and N gene Ct-value, (b) E gene viral load and E gene Ct-value, and (c) RdRp gene viral load and RdRp gene Ct-value.

Examining the association between viral load and cycle threshold (Ct-value) revealed that a lower Ct-value correlates with a higher viral load, suggesting a higher concentration of the viral genetic material in the sample (Figure 3). The correlation between the viral load and the Ct-value reported for the N gene, E gene, and RdRp gene was statistically significant with a strong association of *r* = −0.703 (*p* < 0.001), *r* = −0.931 (*p* < 0.001), and *r* = −0.918 (*p* < 0.001), respectively (Figure 3).

### 4.4. Circulating SARS-CoV-2 Genotypes

**Figure 4:**
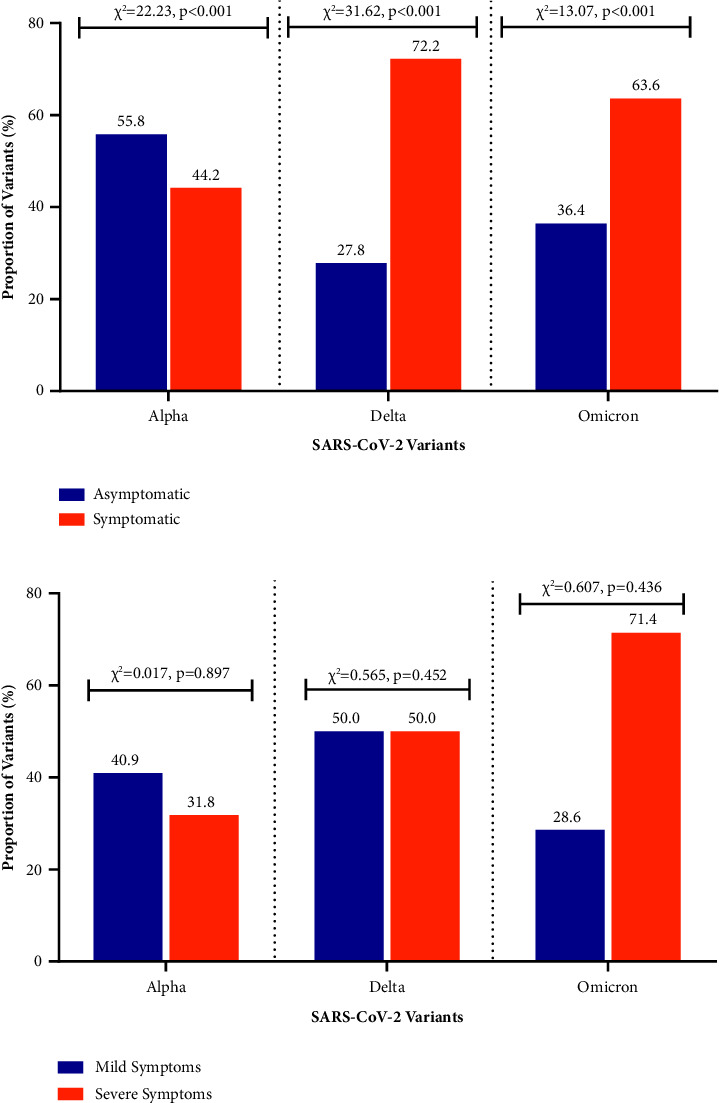
Clinical presentation associated with SARS-CoV-2 variants: (a) symptom status and (b) severity of symptoms.

Three variants of the SARS-CoV-2 were identified among the 81 SARS-CoV-2 positive study participants: Alpha (*n* = 52, 64.2%), Delta (*n* = 18, 22.2%), and Omicron (*n* = 11, 13.6%). The majority of the participants infected with the Omicron (63.6%) and Delta (72.2%) variants were symptomatic, while over 55.8% of the participants infected with the Alpha variant were asymptomatic (Figure 4(a)). The majority of the participants with Omicron-associated SARS-CoV-2 infection presented with severe symptoms (Figure 4(b)).

Furthermore, all the participants infected with the Omicron variants (*n* = 11, 100) of the SARS-CoV-2 had high viral loads (Ct-value <25). More than half (*n* = 10, 55.6%) of the participants infected with the Delta variant (B.1.617.2) also had high viral loads (Table 3). Statistically, there was a significant difference observed between participants infected with the Alpha and Omicron variants (*p* < 0.05) as well as the Alpha and Delta variants (B.1.617.2) (*p* < 0.05) who also had high viral loads (Table 3). Also, there was a significant difference observed between the Alpha and Omicron-infected participants with low viral loads (Table 3).

### 4.5. Risk Factors and Predictors Associated with SARS-CoV-2 Infection

Table 3 gives a detailed description of the risk factors and predictors associated with SARS-CoV-2 infection. Males had an increased risk of SARS-CoV-2 infection (aOR (CI_95_): 0.751, (0.374–1.508)). Similarly, Ghanaians (aOR (CI_95_): 0.822, (0.360–1.877)) and symptomatic (aOR (CI_95_): 35.042, (3.004–108.720)) participants had an increased risk of acquiring SARS-CoV-2. Participants aged 31–40 years (aOR (CI_95_): 2.051, (0.460–9.140)) and 41–50 years (aOR (CI_95_): 2.555, (0.562–11.614)) had higher chances of SARS-CoV-2 infection relative to participants aged 71 years and above. In order of decreasing association with SARS-CoV-2, chills, cough, headache, body weakness, sore throat, and dyspnoea were reported as some of the strong predictors of SARS-CoV-2 infection (Table 4).

## 5. Discussion

### 5.1. Disease Prevalence

The current study determined the prevalence and severity of SARS-CoV-2 and its associated risk factors and predictors of the infection within the Accra Metropolis, postlockdown. The prevalence reported in this study was 30.2% (C I_95_: 24.7–35.8), which is higher than the national cumulative prevalence of 9.7% [13], which is an indication of active transmission of the SARS-CoV-2 virus in the Metropolis. Also, the 13.2% reported by Owusu et al. [13] in the northern belt of Ghana was lower than the prevalence reported in this study. However, the prevalence reported in this study was lower than the 77.9% reported in Turkey [24] and the 62% reported in South Africa [25]. This study's reported prevalence differs from previous studies in Ghana and other countries due to geographical location, environmental conditions, nucleic extraction kits, amplification platforms, and sample size. Variations in epidemiological studies are also accounted for by sample size.

The gender-based difference was noted in this study with the majority of the SARS-CoV-2 infection detected in females (56.8%), although the study involved more males than females. In contrast to a study conducted in Ghana by Owusu et al. [13] and Odikro et al. [2], the prevalence of infection was higher in males (51.5% and 57.8%, respectively) than in females. Furthermore, researchers in China and East Indonesia have also reported a higher number of cases among men [26, 27]. Although females and males have comparable SARS-CoV-2 infection rates, COVID-19 causes more severe symptoms and higher mortality in males than in females, according to available sex-disaggregated epidemiological data [28]. Existing data depict that many biological and behavioural risk factors may have a role in the varied immune responses against SARS-CoV-2 [29].

The study reported that participants aged 61–70 years had a lower incidence of SARS-CoV-2 infection (Table 2). Notwithstanding this, participants aged 31–40 years were observed to have the highest prevalence of SARS-CoV-2 infection (Table 2). The results of the present study varied with the report by Owusu et al. [13] which stated that persons aged 21–30 years had the highest prevalence rate of SARS-CoV-2 infection.

### 5.2. Severity of SARS-CoV-2 Infection

The study assesses SARS-CoV-2 viral load in positive cases, assessing infection intensity and transmission patterns. Comparison of the viral loads between symptomatic and asymptomatic subjects was also performed. Participants presenting with symptoms had higher viral loads than asymptomatic participants (Figure 2(a)). There are mixed reports of variation in viral loads of symptomatic and asymptomatic populations. In a study conducted in Italy, there was no difference in viral loads for symptomatic and asymptomatic subjects [30]. The findings of this study are corroborated with previous reports which observed higher mean viral load in persons with severe symptoms than mild clinical symptoms [13, 31]. Nonetheless, other authors have reported higher viral loads in asymptomatic patients [32, 33], which supports the fact that asymptomatic or minimally symptomatic patients can transmit the virus.

This study reported higher SARS-CoV-2 viral loads in males than in females (Figure 2(b)). The sex difference in viral load was insignificant, and according to previous reports, virus loads in males and females are comparable [34, 35]. However, Mahallawi et al. [36] reported higher viral loads in females than in males, which is consistent with the findings of the present study. It is not surprising to find that sex affects viral load and the immune system's response to an infectious disease; this has been shown to happen with other illnesses. This is assumed to be connected to an immunological response differential, where females acquire a greater immune response to infectious agents, rendering them less susceptible to infections [37].

Similar to the previous studies conducted in Germany [38], Chicago, and the USA [39], higher viral loads were reported in children than in adults (Figure 2(c)). In contrast to this study, Owusu et al. [13] in Ghana reported higher viral loads in adults than in children. Notwithstanding, within the adult age groups, the elderly aged 60 years and above had higher viral loads which is consistent with the findings of Owusu et al. [13]. The findings also indicate that paediatric patients of all ages, from infancy to young adulthood, can carry a high SARS-CoV-2 viral load in their upper airways, particularly early in the course of infection, and an elevated viral load corresponds with high levels of viable, replicating virus [40].

While the findings of this study do support the idea that young children carry a higher viral load, making them more likely to spread SARS-CoV-2 [37–40], we propose an alternative theory for how they contribute to transmission. We believe that children may serve as a reservoir for asymptomatic infections, which could lead to further spread of the virus. It is important to note that while RT-PCR can measure viral load, it cannot distinguish between contagious virions, flawed particles, or lysed cells. In addition, infectiousness can be influenced by a variety of clinical, behavioural, and environmental factors within a population [41]. Ultimately, the only way to confirm an individual's infectiousness is through the culture of respiratory specimens.

### 5.3. SARS-CoV-2 Circulating Genotypes

The findings of this study indicate that SARS-CoV-2 infection within the Metropolis was mainly driven by the Alpha variant (B.1.1.7) during the study period and the same was reported in Ohio [42]. Morang'a et al. [43] indicate in their study that the second wave of SARS-CoV-2 was mainly driven by the Alpha variant (B.1.1.7) but the Delta variant (B.1.617.2) was introduced into the country in May 2021, confirming the findings of this study. In other studies, the Delta variant (B.1.617.2) was the dominant circulating variant [44, 45].

SARS-CoV-2 variants impact clinical presentations and disease severity at varying degrees. Relative to the other two variants identified, the majority of the symptomatic infections were associated with the Delta variant (B.1.617.2) (Figure 4(a)). However, majority of the severe clinical presentations were associated with the Omicron variant (B.1.1.529.2). More so, participants infected with Omicron (B.1.1.529.2)-associated SARS-CoV-2 infection presented with significantly high viral load than the Alpha variant (B.1.1.7) and Delta variant (B.1.617.2) (Table 3). Other studies have also reported that Omicron (B.1.1.529.2) infections are associated with severe forms of infection with reported high viral loads [46, 47] and severe symptoms [48]. The increased infectiousness of the Omicron variant (B.1.1.529.2) stems from immune escape due to altered spike-in antigens [49, 50]. Comparatively, infections associated with the Delta variant (B.1.617.2) show higher viral load and severe symptoms than Alpha variant (B.1.1.7) infections, and this is consistent with the findings of several studies [50]. However, the Alpha variant (B.1.1.7) has the ability to cause breakthrough infections in vaccinated individuals, although the severity of these infections may be lower. Furthermore, it is important to note that the impact of vaccination on the reduction of recovery of infectious viruses is associated with the Alpha variant (B.1.1.7) than the Delta variant (B.1.617.2).

### 5.4. Risk Factors and Predictors Associated with SARS-CoV-2 Infection

The findings of this study indicated that men had an increased risk (Table 4) of SARS-CoV-2 infection, which is consistent with the findings of other studies [13, 51]. Contrarily, researchers in Italy have reported an increased risk of infection in females and an increased risk of SARS-CoV-2 infection [52]. Hormonal response elements, such as AREs and ORE, produce innate immune responses, resulting in dimorphic immunity [53]. Females have higher antibody response, immunoglobulin levels, and B cells, influenced by genetic factors; men are less likely to develop antibodies, higher interferon levels, and higher viral infection susceptibility [54, 55].

Previous studies have reported age to be associated with SARS-CoV-2 infection, principally those aged between 30 and 65 years, with more than half of the cases being older than 40 years [56, 57]. This study reported a similar increased risk of SARS-CoV-2 infection in people between the ages of 30–70, with the highest risk being associated with persons aged between 31 and 40 years (Table 3). Similar to this study, Kostadinova et al. [58] reported an increased risk of infection amongst middle-aged participants, which can be attributed to the social contacts during working and travelling activities, may be the origin of the higher chance of becoming infected in these age groups.

Generally, symptomatic participants were reported to have an increased risk of SARS-CoV-2 infection relative to asymptomatic participants (Table 4). The present study identified cough, headache, sore throat, shortness of breath, and fever (>38°C), as some major predictors of SARS-CoV-2 infection and identified chills as the strongest predictor of SARS-CoV-2 infection amongst the study participants (Table 4). The findings of this study are consistent with previous reports [13, 59] with the exception of anosmia which none of the participants in the present study indicated as a symptom. This varied with several reports that indicated that anosmia was the strongest predictor of SARS-CoV-2 infection [60, 61].

Other studies have indicated that fever and cough were the most prevalent symptoms of SARS-CoV-2 pneumonia at the onset of the infection [62, 63]. Dyspnoea, which is typically seen in sick people with SARS-CoV-2 pneumonia [64], maybe a sign that the disease is progressing and getting worse since it indicates a low oxygenation index. SARS-CoV-2 targets epithelial cells, including pneumocytes, nasal, and bronchial, through ACE2 receptors and TMPRSS2 [65]. SARS-CoV-2 infects endothelial and epithelial cells, enhancing inflammatory response and oxygen diffusion, causing pneumonia-like symptoms due to impaired oxygen transfer [66].

Musculoskeletal involvements in SARS-CoV-2 infection such as body weakness and muscle aches have been reported as predictors of SARS-CoV-2 infection [67] as has been reported in the present study (Table 4). SARS-CoV-2 attaches to skeletal muscle receptors and enters cells through direct virus action through ACE2 and/or TMPRSS2 expression [68]. The indirect mechanism considers musculoskeletal tissue effects from SARS-CoV-2 infection, causing severe inflammation and organ damage, causing symptoms such as weariness and myalgia in symptomatic individuals [68].

Similar to the present study, gastrointestinal tract infection and its symptoms have been reported as predictors of SARS-CoV-2 infection [69]. SARS-CoV-2 infects the gastrointestinal system through ACE2 cell receptors, affecting ACE2 function and causing nausea, diarrhoea, and vomiting due to its control of intestinal inflammation [70].

Limitations of the study include not testing for other well-known respiratory viruses in the participants of this study. This is crucial because people who are symptomatic and have negative results for SARS-CoV-2 could potentially be infected with other respiratory infections.

## 6. Conclusion

The study found a high prevalence of SARS-CoV-2 infection in the Accra Metropolis postlockdown, with most cases reported in females. Male participants had higher infection intensity, while symptomatic participants had a higher disease prevalence and intensity. The highest viral load was found in participants aged 10 years and below, indicating active transmission. The study identified SARS-CoV-2 variants Alpha (B.1.1.7), Delta (B.1.617.2), and Omicron, with high viral loads associated with Omicron infections. Moreover, the study showed that men have a higher risk of infection, while symptomatic participants have an increased risk.

## Figures and Tables

**Table 1:** Demographic characteristics of study participants.

Variable	Categories	*n* (%)	*χ* ^2^	*P* value
Gender	Male	138 (51.5)	0.239	0.625
	Female	130 (48.5)		

Age group (years)	≤10	22 (8.2)	33.33	<0.001
	11–20	27 (10.1)		
	21–30	45 (16.8)		
	31–40	51 (19.0)		
	41–50	41 (15.3)		
	51–60	42 (15.7)		
	61–70	23 (8.6)		
	≥71	17 (6.3)		

Nationality	Ghanaian	213 (79.5)	93.149	<0.001
	Non-Ghanaian	55 (20.5)		

Symptomatic	Yes	55 (20.5)	94.149	<0.001
	No	213 (79.5)		

*n* = number of participants; % = percentage; *χ*^2^ = one sample chi-square. *p* < 0.05 is considered as statistically significant.

**Table 2:** Distribution of SARS-CoV-2 infection among study participants.

Category	Negative (187) (69.8)	Positive (81) (30.2)	Total (268) (100.0)	*P* value
*Gender*				0.075
Male	103 (55.1)	35 (43.2)	138 (51.5)	
Female	84 (44.9)	46 (56.8)	130 (48.5)	

*Age groups (years)*				0.963
≤10	17 (9.1)	5 (6.2)	22 (8.2)	
11–20	20 (10.7)	7 (8.6)	27 (10.1)	
21–30	33 (17.6)	12 (14.8)	45 (16.8)	
31–40	31 (16.6)	20 (24.7)	51 (19.0)	
41–50	24 (12.8)	17 (21.0)	41 (15.3)	
51–60	31 (16.6)	11 (13.6)	42 (15.7)	
61–70	19 (10.2)	4 (4.9)	23 (8.6)	
≥71	12 (6.4)	5 (6.2)	17 (6.3)	

*Nationality*				0.436
Ghanaian	151 (80.7)	62 (76.5)	213 (79.5)	
Non-Ghanaian	36 (19.3)	19 (23.5)	55 (20.5)	

*Symptomatic*				<0.001
Yes	12 (6.4)	43 (53.1)	55 (20.5)	
No	175 (93.6)	38 (46.9)	213 (79.5)	

Values reported are the number of cases and percentages.

**Table 3:** Distribution of SAR-CoV-2 variants according to disease severity.

Cycling threshold	SARS-CoV-2 variants (*n* (%, CI_95_))		
	Alpha	Delta	Omicron
Ct < 25 High viral load	25 (48.1, 46.2–50.0)^a^	10 (55.6, 52.2–59.1)^b^	11 (100.0, 94.2–106.1)^a,b^
25 < Ct < 30 Moderate viral load	9 (17.3, 16.2–18.5)	4 (22.2, 20.1–24.5)	0 (0.0)
30 < Ct < 40 Low viral load	18 (34.6, 33.0–36.3)^c^	4 (22.2, 20.1–24.5)	0 (0.0)^c^
Total	52 (100.0)	18 (100.0)	11 (100.00)

Row values with the same superscript are significant at *p* < 0.05.

**Table 4:** Risk factors and predictors associated with SARS-CoV-2 infection.

Categories	Odds ratio			
	Crude (CI_95_)	*P* value	Adjusted (CI_95_)	*P* value
*Gender* (ref: female)	1		1	
Male	0.62 (0.37–1.06)	0.074	0.5 (0.37–1.51)	0.421

*Age group* (ref: ≥71 years)	1		1	
≤10 years	0.66 (0.26–1.84)	0.424	0.50 (0.07–3.54)	0.490
11–20 years	0.46 (0.17–1.38)	0.161	0.59 (0.10–3.60)	0.565
21–30 years	0.33 (0.17–0.65)	0.001	0.51 (0.10–2.74)	0.432
31–40 years	1.65 (0.90–3.05)	0.120	2.05 (0.46–9.14)	0.346
41–50 years	1.80 (0.92–3.49)	0.089	2.56 (0.56–11.61)	0.225
51–60 years	0.79 (0.37–1.61)	0.536	0.79 (0.16–3.93)	0.776
61–70 years	0.46 (0.17–1.38)	0.161	0.80 (0.13–4.86)	0.809

*Nationality* (ref: non-Ghanaian)				
Ghanaian	0.78 (0.41–1.43)	0.434	0.82 (0.36–1.88)	0.641

*Symptoms status* (ref: asymptomatic)	1		1	
Symptomatic			35.04 (3.00–108.72)	

*Clinical symptoms* (ref: no)	1		1	
Symptomatic	16.50 (7.85–33.70)	<0.001	35.04 (3.00–108.72)	0.005
Chills (yes)	45.79 (7.49–98.65)	<0.001	62.99 (0.96–124.59)	0.052
Cough (yes)	6.03 (1.66–21.89)	0.006	18.11 (8.37–39.53)	<0.001
Diarrhoea (yes)	0.14 (0.01–0.96)	0.049	0.09 (0.001–6.48)	0.271
Fever >38°C (yes)	0.11 (0.02–0.53)	0.002	0.90 (0.11–7.36)	0.925
Nausea (yes)	0.21 (0.02–1.86)	0.167	0.74 (0.02–25.42)	0.866
Dyspnoea (yes)	4.71 (0.54–68.54)	0.167	1.05 (0.03–33.92)	0.980
Sore throat (yes)	11.25 (4.61–27.76)	<0.001	1.50 (0.28–8.12)	0.636
Headache (yes)	16.01 (5.38–44.12)	<0.001	3.32 (0.51–21.51)	0.208
Joint pain (yes)	4.71 (0.54–68.54)	<0.001	0.09 (0.001–5.29)	0.244
Muscle ache (yes)	8.75 (1.87–42.10)	0.002	0.74 (0.03–20.36)	0.859
Runny nose (yes)	7.18 (2.43–20.97)	<0.001	0.37 (0.05–3.06)	0.357
Body weakness (yes)	9.66 (1.55–18.74)	0.014	2.73 (0.03–33.77)	0.659

## Data Availability

The data used to support the findings of the study are included within the article.
